# Editorial for the Special Issue on Implantable Microdevices

**DOI:** 10.3390/mi10090603

**Published:** 2019-09-12

**Authors:** Wen Li, Zhen Qiu

**Affiliations:** 1 Department of Electrical and Computer Engineering, Michigan State University, East Lansing, MI 48824, USA; 2 Department of Biomedical Engineering, Michigan State University, East Lansing, MI 48824, USA; 3 Institute for Quantitative Health Science and Engineering, Michigan State University, East Lansing, MI 48824, USA

Implantable microdevices, providing accurate measurement of target analytes in animals and humans, have always been important in biological science, medical diagnostics, clinical therapy, and personal healthcare. Recently, there have been increasing unmet needs for developing high-performance implants that are small, minimally-invasive, biocompatible, long-term stable, and cost-effective. Therefore, this special issue aims to bring together state-of-the-art research and development contributions that address key challenges and topics related to implantable microdevices.

There are eight research articles and one review article published in this special issue, covering a wide range of topics: implantable sensors for detecting biophysiological and biophysical signals [1,2]; novel transducers for stimulation [3]; miniaturized antennas and biotelemetries for wireless implants [4,5,6] medical electronics and system-on-chip (SoC) [1,7]; and nano/microfabrication technologies for implantable microdevices [2,8,9]. On the device side, Chen et al. reported a tongue pressure sensing platform that integrated interdigitated pressure sensing electrodes with SoC on flexible polymer films. The sensor achieved high sensitivities at the low-pressure region of below 10 kPa and good stability in air and in deionized water. Xi et al. fabricated an ultramicron silver needle electrode (7.9 µm in diameter) using a cost-effective, laser-assisted pulling method [2]. Also published is a new piezoelectric transducer implant proposed by Liu et al. for stimulation of the round window membrane of the middle ear with significantly reduced power consumption and improved output quality [3]. On the system side, Lee et al. presented a highly efficient, wireless ECG monitoring system that integrated ECG sensor, temperature sensor, microcontroller unit, radio-frequency transceiver, and wireless power transmission unit on a single printed circuit board for continuous monitoring of cardiac arrhythmias [7].

Inductive telemetries have been widely used in biomedical implants for wireless power delivery and data communication. This special issue includes multiple designs of wireless antennas that have been explored by different groups. In particular, Hajiaghajani et al. proposed a three-dimensional coil array based on a generalized Halbach array for wireless powering of biomedical instruments within a large area of 144 cm^2^ while eliminating the need for traditional magnetic shielding [4]. In another design, Fan et al. designed a miniaturized circularly polarized antenna for in-body wireless communication with broadened bandwidth [5]. Li et al. also proposed a compact broadband implantable antenna with dual resonance bandwidth of 52 MHz at low return loss of −10 dB. Packaging and surface fouling are also long-standing challenges of biomedical implants. Zeng et al. presented a comprehensive review article that summarizes the micro/nanotechnologies and material considerations for high-density implantable packaging and flexible microelectrode arrays used in artificial retinal prostheses [8]. To reduce the surface fouling effect, Qin et al. proposed a dual bio-inspired shark-skin and lotus-structure surface with a unique combination of superhydrophobic, antidrag, and self-cleaning properties [9]. 

We would like to take this opportunity to thank all the authors for submitting their papers to this special issue. We also want to thank all the reviewers for dedicating their time and helping to improve the quality of the submitted papers.
